# Association between Long COVID and Overweight/Obesity

**DOI:** 10.3390/jcm10184143

**Published:** 2021-09-14

**Authors:** Luigi Vimercati, Luigi De Maria, Marco Quarato, Antonio Caputi, Loreto Gesualdo, Giovanni Migliore, Domenica Cavone, Stefania Sponselli, Antonella Pipoli, Francesco Inchingolo, Antonio Scarano, Felice Lorusso, Pasquale Stefanizzi, Silvio Tafuri

**Affiliations:** 1 Interdisciplinary Department of Medicine, Section of Occupational Medicine, University of Bari, 70124 Bari, Italy; luigi.demaria@uniba.it (L.D.M.); marcoquarato.uniba@gmail.com (M.Q.); antonio.caputi@uniba.it (A.C.); domenica.cavone@uniba.it (D.C.); stefania.sponselli@uniba.it (S.S.); antonella.pipoli@uniba.it (A.P.); 2 Occupational Medicine Unit, University Hospital of Bari, 70124 Bari, Italy; 3 School of Medicine, University of Bari, 70124 Bari, Italy; loreto.gesualdo@uniba.it; 4 General Direction, University Hospital of Bari, 70124 Bari, Italy; giovanni.migliore@policlinico.ba.it; 5 Interdisciplinary Department of Medicine, Section of Dental Medicine, University of Bari, 70124 Bari, Italy; francesco.inchingolo@uniba.it; 6 Department of Innovative Technologies in Medicine and Dentistry, University of Chieti-Pescara, 66100 Chieti, Italy; 7 Department of Biomedical Science and Human Oncology, University of Bari, 70124 Bari, Italy; pasquale.stefanizzi@uniba.it (P.S.); Silvio.tafuri@uniba.it (S.T.)

**Keywords:** 35-day long-COVID, risk factors, overweight, obesity, respiratory diseases, healthcare workers

## Abstract

Background: Long COVID is a syndrome characterized by the persistence of SARS-CoV-2 infection symptoms. Among HCWs, prolonged COVID symptoms could lead to the inability to perform work tasks. The aim of this study is to investigate 35-day long-COVID (35-LC) characteristics and risk factors in a one-year period. Methods: We carried out a retrospective cohort study during the COVID-19 pandemic at University Hospital of Bari. A total of 5750 HCWs were tested for close contact with a confirmed case, in the absence of personal protective equipment, or for symptom development. Results: Each positive HCW was investigated for cardiovascular risk factors or respiratory diseases. An amount of 352 HCWs (6.1%) were infected by SARS-CoV-2, and 168 cases evolved to long COVID. The 35-LC group showed mean BMI values higher than the non-35-LC group (25.9 kg/m^2^ vs. 24.8 kg/m^2^, respectively), and this difference was significant (*p*-value: 0.020). Moreover, HCWs who suffered from pulmonary disease (OR = 3.7, CL 95%: 1.35–10.53; *p*-value = 0.007) or overweight (OR = 1.6 CL 95%: 1.05–2.56; *p*-value = 0.029) had an increased risk of developing 35-LC. Conclusions: Long COVID is an emerging problem for hospital managers as it may reduce the number of HCWs deployed in the fight against COVID-19. High BMI and previous pulmonary disease could be risk factors for 35-LC development in exposed HCWs.

## 1. Introduction

Long COVID has been defined as the persistence of symptoms, or the development of new symptoms, relating to SARS-CoV-2 infection late in the course of COVID-19, at least 28 days after diagnosis. Symptom frequency could be constant or fluctuate, and it may involve other systems [1]. Studies have shown that organ involvement in acute disease is not random, but rather probably driven by angiotensin converting enzyme receptor 2 (ACE-R2) distribution in target cells [2], whereas little information is available about the pathogenic association between long COVID-19 and symptom persistence after a negative swab test.

In this regard, SARS-CoV-2 infection is known to lead to late infection and/or post-infection damage to the central nervous system. The brain can be affected, with post-acute viral encephalitis [3], but so can the spinal cord, with inflammation or infarction [4]. In these cases, a prolonged inflammatory response, high interleukin-6 levels and a negative RT-PCR test are demonstrated and could explain the clinical findings [5]. Moreover, in other organs, symptoms may be linked to fibrosis induced by cytokine storms in the acute phase, specifically chronic lung and heart damage demonstrated by flow reduction in spirometry tests and persistent elevated levels of troponin T (TNT) and brain natriuretic peptide (BNP). These are thought to be due to transforming growth factor (TGF)-beta-related fibrosis remodeling in the chronic phase, with typical findings in other diagnostic tests (sonography or chest CT) [6]. Fibrosis-related organ dysfunction is likely associated with high levels of proinflammatory cytokines (interleukin 1, tumor necrosis factor alpha, and interleukin 6) in the acute phase, suggesting that severe exudate in these organs can lead to the deposition of collagenous matrix [7].

However, a fraction of patients affected by SARS-CoV-2 infection, after the acute phase, continue to experience the persistence of typical COVID-19 symptoms, particularly dyspnea, tachycardia and extreme fatigue, despite inflammation index normalization and unremarkable diagnostic test values [8]. Other patients experience a set of muscle-related and psychopathological symptoms related to intense distress (i.e., post-traumatic stress disorder, secondary traumatic stress, complicated grief and anxiety, myalgia, muscle fatigue amongst others). This corollary of symptoms is particularly exacerbated within frontline healthcare workers (HCWs), a category of professionals who are at a significant risk of experiencing high levels of burnout and compassion fatigue. Moreover, autonomic nervous system [9] dysfunction (characterized by night sweats, temperature dysregulation, gastroparesis, constipation/loose stools and peripheral vasoconstriction) was also described and seems to have been more frequent in HCWs during the first wave of the pandemic for those who were exposed to high viral loads; however, no peer-reviewed studies on this topic are yet available [10]. Finally, long COVID seems to be a puzzle of multiple syndromes after the acute infection phase [11].

Based on these findings, long COVID syndrome, both inflammation- and non-inflammation-mediated, could lead to an inability to perform daily home and work tasks. In the healthcare setting, this point is crucial to avoid a reduction in the number of HCWs deployed during pandemic resurgence, in addition to adequate SARS-CoV-2 transmission protection and prevention measures to address the problem.

To date, few studies are available that are related to predicting the evolution of COVID-19 to long COVID syndrome. In particular, Sudre et al. [12] found that age, sex and first-week symptom characteristics could be useful to distinguish patients who experience a short (less than ten days) course of infection (<10 d COVID) from at least 28 day-symptomatic people (28-long COVID or 28-LC), but there are no data available regarding patients who are symptomatic for longer than 28 days.

After the application of strict contagion prevention measures [13,14,15,16] in Bari University Hospital, Southern Italy, our research team performed an exploratory study to identify the characteristics and risk factors of 35-day long-COVID (35-LC) subjects registered between 8 March 2020 and 15 March 2021.

## 2. Materials and Methods

We established a one-year retrospective cohort study of HCWs working at the University Hospital of Bari who were infected by SARS-CoV-2 in this single-center setting. In this study cohort, physicians, nurses and social health assistants (SHAs) were included after at least one nasopharyngeal swab test (NST) with a positive molecular assay result, independent of exposure type (occupational or not). The collection of nasopharyngeal swab specimens, taken by trained staff, followed adequate standard operating procedures (SOPs), and during collection, all specimens were handled carefully according to World Health Organization (WHO) criteria [17,18]. The specimens underwent nucleic acid amplification tests (NAATs) for COVID-19. This method is based on the detection of unique sequences of viral RNA by real-time reverse transcription polymerase chain reaction (rRT-PCR). HCWs in different departments were submitted to NST after, in the first pandemic phase, experiencing close contact without PPE (personal protective equipment), in either an occupational or non-occupational setting, or any SARS-CoV-2 infection symptoms in the following pandemic waves. Every HCW with pending NST results was removed from the workplace, as were any colleagues who had close contacts with symptomatic HCWs.

All HCWs underwent routine health surveillance according to the Italian regulation, which includes physical examination, laboratory tests and instrumental tests (spirometry and electrocardiography). Each positive HCW also received a medical examination according to regulation and protocols on the reintegration into the workplace of subjects who have overcome the SARS-CoV-2 infection. Careful review of medical history was performed to document cardiovascular risk factors (body mass index, systolic and diastolic blood pressure, total and fractioned cholesterol levels, triglyceride levels and fasting glucose levels) and the presence of respiratory disease.

Return to work was possible after at least one negative NST; however, HCWs with negative NST but who were still symptomatic did not resume work.

To carry out this exploratory study, descriptive and inferential analyses were run. For continuous variables, the results are expressed as the mean and standard deviation (S.D.). The Shapiro–Wilk test, for the assessment of distribution normality, was performed [19]. The variables were not distributed normally, so comparisons between groups of interest were formally conducted with the Mann–Whitney test, and the correlation study was carried out using the Spearman test. For categorical variables, the results were expressed as absolute frequencies and percentages, and comparisons between groups of interest were formally conducted with the chi-square test and with Fisher’s exact test when appropriate. Odds ratios and 95% confidence intervals were also calculated to assess the effect of the risk factors [20]. As the correlation between the continuous variables was not significant, no linear regression models were performed. Differences were considered significant if *p* < 0.05. Data were analyzed using SPSS Statistics Version 26 (International Business Machines—IBM).

## 3. Results

During the one-year observation period, 352 of 5750 HCWs (6.1%) were infected by SARS-CoV-2. Specifically, 148 (42%) were males, 204 (58%) were females and the mean age was 45 years (y) (SD = 13.2 y, range = 20–73 y). These HCWs and their close contacts remained away from work until a negative swab test was obtained. Regarding confirmed cases, 21 of 352 patients suffered from pulmonary diseases such as obstructive sleep apnea syndrome (OSAS), chronic obstructive pulmonary disease (COPD) and bronchial asthma (6%); 63 suffered from hypertension (17.9%) and 141 (40.1%) had a body mass index (BMI) > 25 kg/m^2^. In particular, 47 were affected by overweight (25 ≤ BMI < 30 kg/m^2^) and 19 were affected by obesity (BMI ≥ 30) (Table 1).

The most frequent symptoms experienced by HCWs were fever >37.5 °C (58.5%), myalgia and/or asthenia (48.3%) and anosmia (39.8%). Of the 352 HCWs, only 3 (0.8%) required hospitalization.

In the whole sample of infected HCWs, the mean BMI value was 25.4 kg/m^2^ (SD = 4.5 kg/m^2^; range 16.5–48.2 kg/m^2^); mean systolic blood pressure (SBP) and diastolic blood pressure (DBP) values were 123.5 mmHg (SD = 15.2 mmHg) and 78.5 mmHg (SD = 9.7 mmHg), respectively; mean total, HDL and LDL cholesterol blood values were, respectively, 5.05 mmol/L (SD = 0.93 mmol/L), 1.6 mmol/L (SD = 0.45 mmol/L) and 2.98 mmol/L (SD = 0.83 mmol/L). The mean triglyceride value was 1.1 mmol/L (SD = 0.69 mmol/L), and the mean fasting glucose value was 4.73 mmol/L (SD = 0.88 mmol/L) (Table 2).

Finally, the average number of job absence days was 39.1 (SD: 21.6 days; range 3–146 days). HCWs who were absent for more than 34 days were classified as 35-day-long COVID (35-LC); the number of HCWs with 35-LC was 168 (47.7%) and the number without was 184 (52.3%).

The not-35-LC group included 77 (41.8%) males and 107 (58.1%) females, and the 35-LC group included 71 (42.2%) males and 97 females (57.7%); no significant difference was found between the two groups (*p*-value: 0.937). Thirty (16.5%) HCWs in the non-35-LC group suffered from hypertension, while 33 (20.4%) HCWs in the 35-LC group suffered from hypertension, and this difference was not significant (*p*-value: 0.352).

In the 35-LC group, 16 HCWs (9.5%) were affected by respiratory disease, whereas in the non-35-LC group, this number was 5 (2.7%), and this difference was significant (*p*-value: 0.007). In addition, in the 35-LC group, 75 (51%) HCWs had BMI > 25 kg/m^2^, whereas in the non-35-LC group, 66 HCWs (38.8%) met this criterion, and this difference was significant (*p*-value: 0.029) (Table 3).

Thus, HCWs with pulmonary disease (OSAS, BPCO and bronchial asthma) or with overweight/obesity had an increased risk of developing 35-LC instead of a sub-Acute/Early Phase COVID-19 (OR = 3.7, CL 95%: 1.35–10.53; *p*-value: 0.007; OR = 1.6, CI 95%: 1.05–2.56; *p*-value: 0.029). Our data suggest that being affected by overweight or obesity is associated with prolonged symptoms after resolution; the 35-LC group showed a higher mean BMI value than the non-35-LC group (25.9 kg/m^2^ vs. 24.8 kg/m^2^, respectively), and this difference, although small, was statistically significant (*p*-value: 0.020). Age, blood pressure, cholesterol, triglycerides and fasting glucose were not significantly different between the two groups (Table 4).

Moreover, the frequency of subjects with autoimmune, cardiovascular, endocrinological and neurological diseases in the 35-LC group compared to the non-35-LC group was not significantly different.

## 4. Discussion

Long COVID is an emerging problem for hospital managers because of the possible reduction in the numbers of HCWs deployed in the fight against COVID-19. Long absences from work could pose challenges to the organization in terms of staffing work shifts, and it can cause the few remaining HCWs to become exhausted from overwork. On this basis, the identification of particularly susceptible HCWs could be helpful to specifically protect them from infection risk. However, correct contagion prevention measures should be carefully adopted by all HCWs because the greatest risk factor is suboptimal hand hygiene, as demonstrated in hospital settings in Wuhan [21] and worldwide [22,23,24,25,26].

SARS-CoV-2 infection causes a cascade effect in humans; a flu-like syndrome can trigger life-threatening bilateral pneumonia, whose symptoms could last for several weeks. In this regard, Nalbandian and colleagues concluded, based on a recent review of the literature, that long COVID could be distinguished from early-phase or subacute COVID-19 (from 4 to 12 weeks) and chronic COVID-19 (which includes symptoms and abnormalities persisting or present beyond 12 weeks from the date of onset of acute COVID-19 and is not attributable to alternative diagnoses); in both syndromes, isolation of the virus from the respiratory tract and/or nasopharyngeal swabs is unlikely later than 4 weeks after initial onset, suggesting post-infective mechanisms [27].

Our data suggest that being affected by overweight or obesity is associated with prolonged symptoms after resolution.

Among 5750 HCWs in a one-year period of observation, 199 (3.46%) developed 35-LC; in this group, 24 suffered from pulmonary disease, and 88 had a BMI > 25 kg/m^2^. However, the comparison of instrumental and functional respiratory tests, performed in these HCWs prior and after the COVID-19 disease, showed no difference in the pulmonary and cardiac conditions. These findings could be partially explained by the hypothesis of the autonomic symptom origin (because high catecholamine levels can lead to paradoxical vasodilatation, sympathetic activity withdrawal and activation of the vagus nerve) [28] or posttraumatic stress disorder (PTSD), identifying dyspnea and fatigue as anxiety equivalents, which were also found in other small cohort studies [29,30].

In our study, other variables (SBP, DBP, cholesterol and triglyceride levels) had no associations with long COVID onset, suggesting that classic cardiac risk factors and cardiovascular physiopathology were unable to describe the etiopathogenesis of these long-term symptoms.

However, in our study, BMI > 25 kg/m^2^ (another classic cardiovascular risk factor) was more frequent in the 35-LC group; in this regard, several meta-analyses showed that obesity is strongly associated with COVID-19 development and that BMI > 35 kg/m^2^ was an independent risk factor for mortality from COVID-19 [31]. Consequently, individuals affected by overweight and obesity should be subjected to targeted active health surveillance. A BMI of more than 25 kg/m^2^ could be used as a level of attention in the clinical setting. The results of this exploratory study may bring further studies to identify the actual level of risk of long COVID syndrome in a population stratified by BMI classes.

A limitation of this study is that the population is composed exclusively of HCWs subjected to a high clinical-instrumental screening and serological and molecular swab testing [32]. Another limitation is the lack of data on waist circumference, lean body mass and fat mass of HCWs. Moreover, the 168 HCWs do not represent a very large sample of 35-LCs. On the one hand, this is a limitation of our study, on the other it is a strength in regards to the effectiveness of the application of prevention and protection measures in a pandemic context. Furthermore, in the near future, the reported data will be related to the benefits of the current vaccination campaign [33].

Starting from these reference points, our data suggest that overweight and obesity [34] could lead to prolonged symptoms after resolution of SARS-CoV-2 infection, but further investigation, examining both autonomic nervous system dysfunction and the clinical features attributable to PTSD, is needed to identify the exact pathogenic pathway.

## 5. Conclusions

Long COVID is an emerging problem for the clinical work setting, which is due to the possible reduction in the numbers of available employees in all production sectors and engagement in the fight against COVID-19; in this regard, the ability to identify susceptible HCWs who are more likely to experience persistent chronic post-viral infection symptoms is crucial to preserve the correct staffing of work shifts. In our study, we found that among HCWs, those with respiratory problems or BMI > 25 kg/m^2^ had an increased risk of developing long COVID, with symptoms lasting for at least 35 days. It would therefore be necessary to raise awareness on the importance of prevention policy and health promotion activities, especially during the health surveillance of workers. In this regard, lifestyle interventions can prevent the adverse outcome of several diseases, as well as complications, in case of diseases such as COVID-19.

Our findings also represent a valid starting point for future clinical research on people with high risk of complications in case of COVID-19. Finally, identifying classes of frail people could also be important to guide the next vaccination strategies and the development of innovative drug therapies.

## Figures and Tables

**Table 1:** The frequency of subjects with respiratory diseases, hypertension, BMI > 25 kg/m^2^ and 35-LC in all subjects.

All Subjects (*n* = 352)		
	*n*	%
Respiratory diseases	21	6.0
Hypertension	63	17.9
BMI > 25 (kg/m^2^)	141	40.1

**Table 2:** Mean age, BMI and hematochemical parameters of all subjects.

All Subjects (*n* = 352)		
	Mean	SD
Age (years)	45.4	13.2
BMI (kg/m^2^)	25.4	4.5
SBP (mmHg)	123.5	15.2
DBP (mmHg)	78.5	9.7
Total cholesterol (mmol/L)	5.05	0.93
HDL cholesterol (mmol/L)	1.6	0.45
LDL cholesterol (mmol/L)	2.98	0.83
Triglycerides (mmol/L)	1.1	0.69
Fasting glucose (mmol/L)	4.73	0.88

**Table 3:** Comparison of the frequencies of respiratory diseases, hypertension and BMI > 25 kg/m^2^ between non-35-long COVID and 35-long COVID groups.

Non-35-LC vs. 35-LC Groups					
	not-35-LC (*n* = 184)		35-LC (*n* = 168)		
	*n*	%	*n*	%	*p*
Respiratory diseases	5	2.7	16	9.5	0.007
Hypertension	30	16.5	33	20.4	0.352
BMI > 25 kg/m^2^	66	38.8	75	51	0.029

**Table 4:** Comparison of mean age, BMI, blood pressure and hematochemical parameters between non-35-Long-COVID and 35-Long-COVID patients.

Not-35-LC vs. 35-LC Groups					
	not-35-LC (*n* = 184)		35-LC (*n* = 168)		
	Mean	s.d.	Mean	s.d.	*p*
Age (years)	44.2	13.2	45.2	13.3	0.359
BMI (kg/m^2^)	24.8	4.2	25.9	4.9	0.020
SBP (mmHg)	122.4	15.8	124.1	15.2	0.587
DBP (mmHg)	77.8	9.7	79.1	9.9	0.167
Total cholesterol (mmol/L)	5.05	0.92	5.06	0.96	0.658
HDL cholesterol (mmol/L)	1.61	0.46	1.59	0.46	0.836
LDL cholesterol (mmol/L)	2.97	0.81	3.02	0.89	0.583
Triglycerides (mmol/L)	1.07	0.64	1.11	0.65	0.269
Fasting glucose (mmol/L)	4.63	0.55	4.83	1.12	0.505

## Data Availability

The data presented in this study are available on request from the corresponding author.
